# Using ontologies to study cell transitions

**DOI:** 10.1186/2041-1480-4-25

**Published:** 2013-10-08

**Authors:** Georg Fuellen, Ludger Jansen, Ulf Leser, Andreas Kurtz

**Affiliations:** 1 Institute for Biostatistics and Informatics in Medicine and Ageing Research, Rostock Medical School, Ernst-Heydemann-Str. 8, 18057 Rostock, Germany; 2 Institute of Philosophy, University of Rostock, 18051 Rostock, Germany; 3 Humboldt Universität zu Berlin, Unter den Linden 6, 10099 Berlin, Germany; 4 Charite Berlin, BCRT, Augustenburger Platz 1, 13353 Berlin, Germany; 5 College of Veterinary Medicine and Research Institute for Veterinary Science, Seoul National University, Seoul, Republic of Korea; 6 Department of Philosophy, University of Münster, Domplatz 6, 48143 Münster, Germany

## Abstract

**Background:**

Understanding, modelling and influencing the transition between different states of cells, be it reprogramming of somatic cells to pluripotency or trans-differentiation between cells, is a hot topic in current biomedical and cell-biological research. Nevertheless, the large body of published knowledge in this area is underused, as most results are only represented in natural language, impeding their finding, comparison, aggregation, and usage. Scientific understanding of the complex molecular mechanisms underlying cell transitions could be improved by making essential pieces of knowledge available in a formal (and thus computable) manner.

**Results:**

We describe the outline of two ontologies for cell phenotypes and for cellular mechanisms which together enable the representation of data curated from the literature or obtained by bioinformatics analyses and thus for building a knowledge base on mechanisms involved in cellular reprogramming. In particular, we discuss how comprehensive ontologies of cell phenotypes and of changes in mechanisms can be designed using the entity-quality (EQ) model.

**Conclusions:**

We show that the principles for building cellular ontologies published in this work allow deeper insights into the relations between the continuants (cell phenotypes) and the occurrents (cell mechanism changes) involved in cellular reprogramming, although implementation remains for future work. Further, our design principles lead to ontologies that allow the meaningful application of similarity searches in the spaces of cell phenotypes and of mechanisms, and, especially, of changes of mechanisms during cellular transitions.

## Background

The (artificial) induction of cell transitions has recently attracted a lot of attention. A cell phenotype (or cell type) can be defined by the cell’s repertoire of molecules and structural components at a certain time, together with the specific morphology and function they bring with them. A cell transition is a change in a cell that results in a new phenotype. For example, the phenotype of epithelial cells is distinct from the phenotype of fibroblasts. Programming of cells is the induction of a cell phenotype transition, e.g. from fibroblast to epithelial cell. Reprogramming is the artificially induced transition of a cell to a cell phenotype, which it (or its predecessor) had in the past. Potency can be defined as the disposition of a cell to transition into another cell phenotype; pluripotency is the ability of a cell to transition naturally into any of the cell phenotypes of an organism (where a transition is natural if it is not triggered by a technical intervention). Since Takahashi and Yamanaka described cell reprogramming of fibroblasts back to pluripotency (also known as generation of iPS, induced pluripotent stem cells) [1], hundreds of papers have dissected the reprogramming process and the cellular disposition of pluripotency at an ever-increasing resolution, reviewed in, e.g., [2] and [3]. This corpus is currently underused as there is no formal representation of the reported findings.

Several ontologies already exist in the domain of cell biology, such as the well-known Gene Ontology (GO) [4] and the cell type ontology (CL; cf. [5,6]). Bard et al. [5] proposed formal definitions for CL classes, referring to properties of cells such as expressed proteins, activated biological processes, or phenotypic characteristics. Further cell-related knowledge projects include the Virtual Physiological Human project (http://www.vph-noe.eu/) that attempts to provide interoperability between different databases and tools related to human physiology and gene expression; the associated software Phenomeblast (code.google.com/p/phenomeblast) is an ontology-based tool for aligning and comparing phenotypes across species. However, many efforts in formal modelling of biological phenomena of organisms focus on anatomical features and only rarely address the cell level (cf. [7-10] and [11]). What is missing is a comprehensive tool to represent and to compare cellular phenotypes and their dynamics.

## Results and discussion

### Cell phenotypes and cell mechanisms

**Figure 1:**
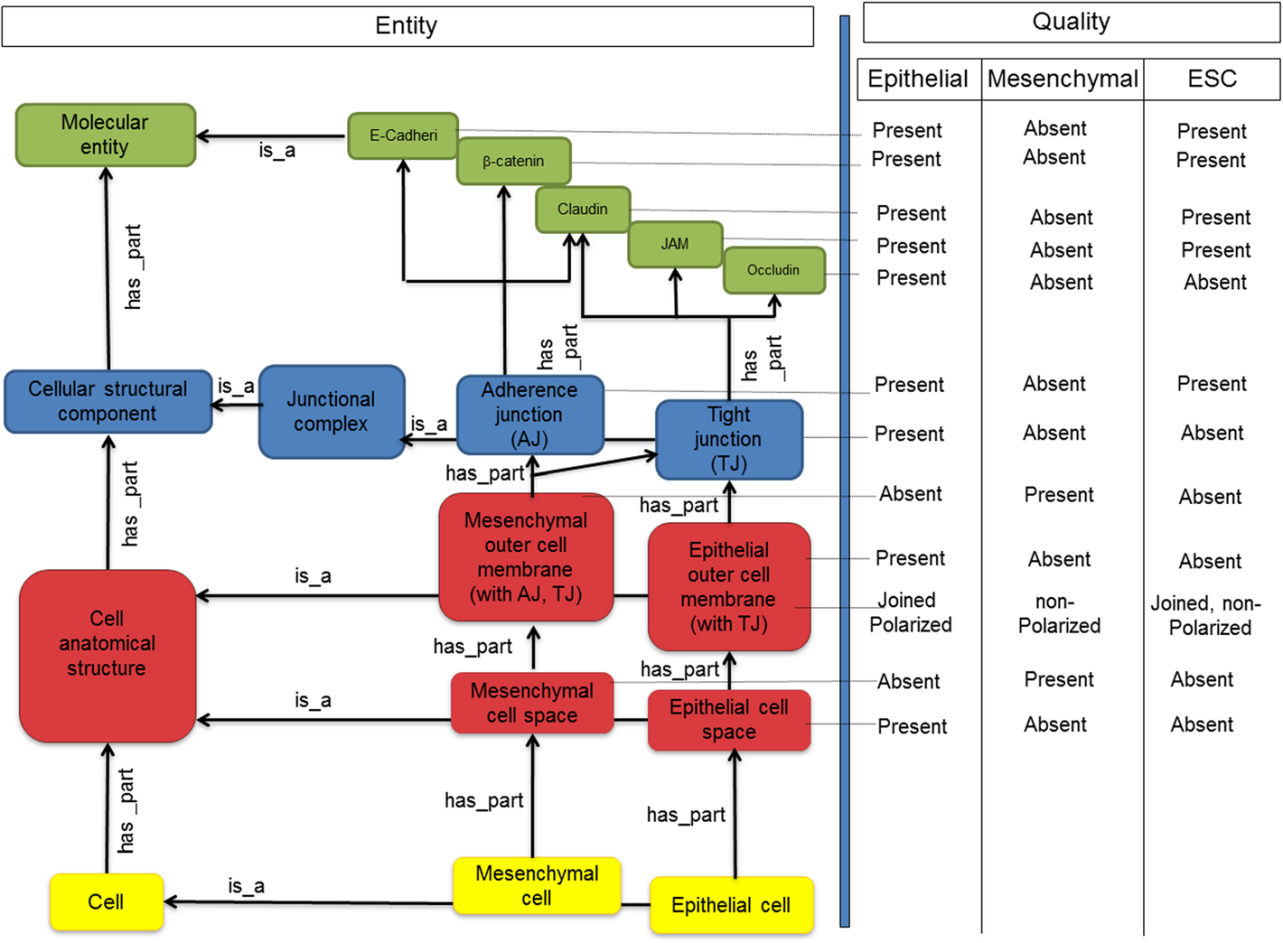
**Outline of an ontology of cell parts and its use to describe cell phenotypes.** The figure shows a structure by which cell phenotypes, here for epithelial cells, mesenchymal cells and embryonic stem cells (ESC), can be formally represented, using entity terms (shown on the left hand side) and PATO-analogous quality modifiers (shown on the right hand side). Terms referring to cells are indicated in yellow, terms relating to structures in red, to ultrastructures in blue, and to molecules in green. With the exception of “is_a”, all relations are meant to have an all-some syntax, i.e. “Tight junction has_part Occludin” means: For *all* instances x of the type Tight junction there is *some* y that is an instance of the type Occludin such that x has part y.

**Figure 2:**
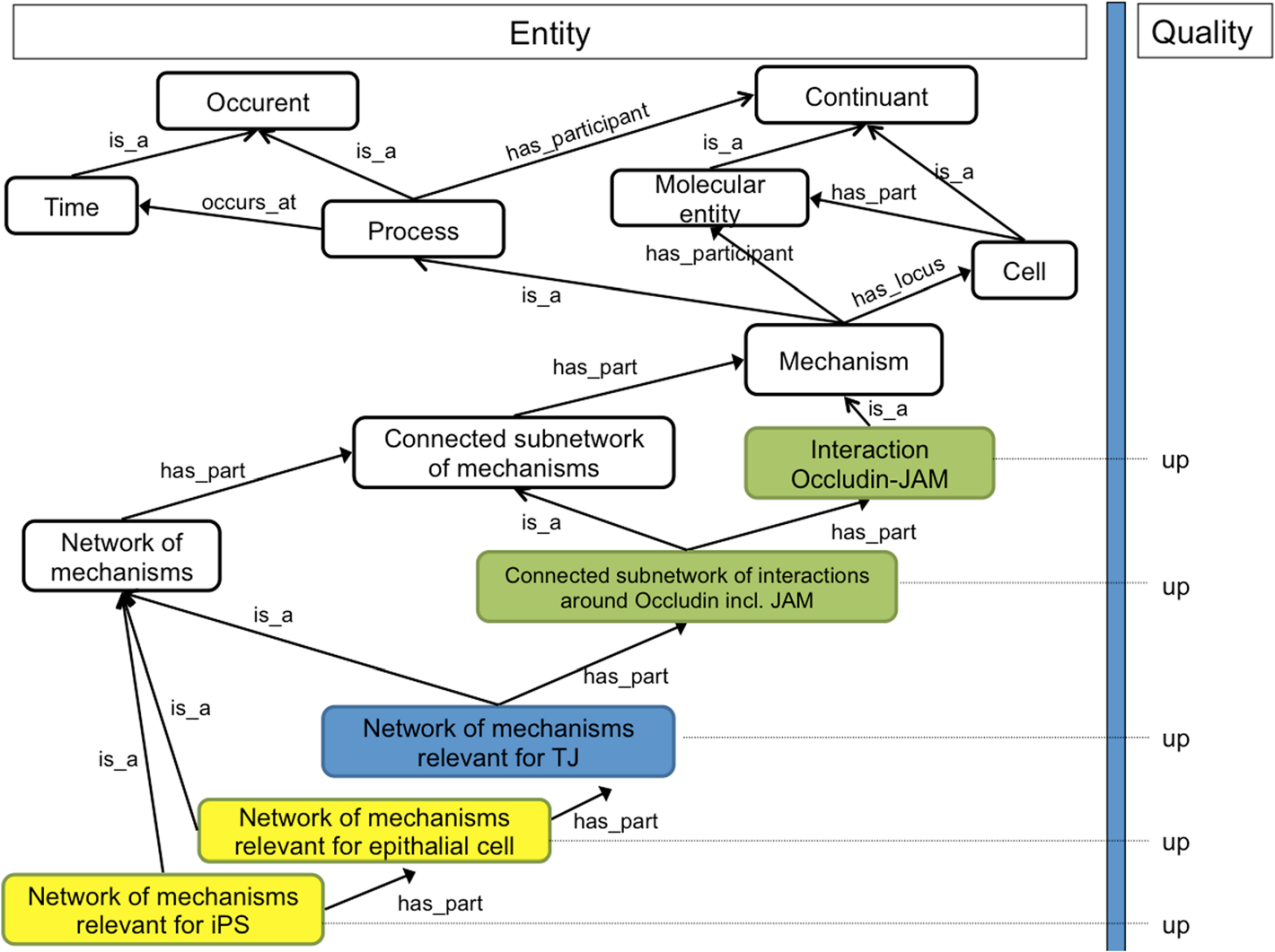
**Outline of an ontology of cell mechanisms and its use to describe cell transitions.** The figure shows a structure by which mechanism changes can be formally represented, using entity terms (shown on the left hand side) and quality modifiers (shown on the right hand side). The colour code follows the code used in Figure 3: Occurrents relevant for cell phenotypes are indicated in yellow, occurrents relevant for ultrastructures in blue, and occurrents directly involving molecules in green. 'Up’ and 'down’ are intended to indicate relative changes: 'Interaction Occludin-JAM Up’ states that there is a development in the cell to feature more interactions of this kind, no matter how many of them there have been before. With the exception of “is_a”, all relations are meant to have an all-some syntax (cf. Figure 1).

We distinguish between two types of processes going on in a cell: microscale mechanisms and macroscale changes thereof. Microscale mechanisms are the interactions between molecules going on in a cell at a certain time, while a macroscale change is the transition from one set of microscale mechanisms going on at one point of time to another such set at a later time. In order to transfer ontology-based annotation and search strategies from phenotypes at the anatomical level [12] to the domain of cell phenotypes and mechanism changes, we need to be able to formally describe both (a) cell phenotypes and (b) mechanism changes. Phenotypes are usually described by means of the entity-quality syntax (EQ) using the Phenotypic Quality Ontology PATO for anatomic phenotypes [13,14]. To apply the EQ syntax to the cell level, we outlined two ontologies, an ontology of cell parts (Figure 1) and an ontology of microscale mechanisms (Figure 2) to be used in combination with a small set of standardized modifiers (as 'qualities’).

Most of the classes that are needed for the ontology of cell parts can be found in CELDA, the ontology developed by the CellFinder project (http://cellfinder.org/about/ontology/) [15], that itself integrates ontologies like the Cell Ontology (CL), the Cell Line Ontology (CLO), the Foundational Model of Anatomy (FMA), the Gene Ontology (GO) and Mouse Anatomy. Additional classes can be taken from the Cellular Phenotype Ontology [16] (CPO). For the ontology of cell mechanisms, we can re-use (portions of) the Interaction Network Ontology (http://bioportal.bioontology.org/ontologies/1515) and the GO subontology for biological processes (http://www.geneontology.org). The current GO (Biological Process), however, does not capture the hierarchical relationships described in Figure 2, connecting molecular events such as the interaction of Occludin and JAM to ultrastructural events such as the formation of a tight junction. Here, we need to explicitly encode the interconnections of molecular events and ultrastructural events. The ontology of cell parts (Figure 1) is designed to handle exactly the same challenge, on the level of the continuants. While the focus of the CPO is on phenotype *abnormalities*, we can still re-use it to provide distinct morphological and associated physiological phenotypes of cells and their components. Again, the hierarchical interconnections between the molecular entities and cellular parts (components, anatomical structures, cell types) need to be explicitly established, for example, between Occludin in tight junctions to the anatomy of specific cell types.

To describe cell phenotypes and transitions, we refer to entities belonging to distinct ontological top-level categories [17]:

(1) Independent continuants: Cells and their organelles as well as molecules are three-dimensional entities; they are present with all their spatial parts at every time of their existence.

(2) Dependent continuants: Any property of a cell or a molecule, be it a quality or a disposition, also exists as a whole at every time of its existence. However, any such property is ontologically dependent on its particular bearer: It cannot exist without it.

(3) Occurrents: Interactions, inhibitions, stimulations as well as transitions are temporally extended processes. They have temporal parts that occur at different times; hence they do not exist as a whole at any single point of time.

We can, for example, describe the state of a cell at a certain time by enumerating all of its parts and contents (independent continuants), or by enumerating all of its properties (dependent continuants), or by enumerating all the events going on at this time (occurrents), which could then be connected with parts and contents of the cell as their participants, e.g. with organelles or molecules. All of these categories are needed to integrate the data available: Cellular data describe continuants (like cellular components and dispositions) as well as occurrents, namely the molecular interactions (microscale mechanisms) going on in a cell at a certain time. While these continuants are covered by the phenotype ontology scheme, the interactions (microscale mechanisms) are covered by the mechanism ontology. Cell transition data describe occurrents, namely macroscale changes of microscale mechanisms. Within the EQ framework, we can describe such macroscale changes of microscale mechanisms by pairing terms for microscale mechanisms (as 'entities’) with specific change modifiers (as 'qualities’). In Figure 2 we illustrate this with one possible annotation pattern for a cell transition. In this annotation pattern, the entity term 'Interaction Occludin-JAM’ from the mechanism ontology is used as a subject term in combination with the qualifier 'up’ in order to express that in a certain time step the interaction between Occludin and JAM has been upregulated.

In our framework, a pluripotent cell can then be characterized by its expression data (about genes, proteins etc.), from which relevant microscale mechanisms can be inferred. A cell transition from one cell phenotype into another (e.g., of a fibroblast into a pluripotent cell) can be described by comparing the expression data of both cell phenotypes, which capture macroscale changes in microscale mechanisms. Such expression data include the start-up of the interactions between genes/proteins relevant for the induction of pluripotency. Such a start-up may happen because the cell starts to produce more instances of the molecule types participating in this type of interaction. In our framework, a pluripotent cell realizes dispositions for mechanisms relevant for pluripotency that may be described by a network of interactions. Further, a cell transition from fibroblast to pluripotent cell realizes dispositions for changes in mechanisms. After transition, the cell is characterized by the microscale mechanisms relevant for the pluripotent phenotype.

### The use of the ontologies within the EQ framework

Our ontologies are designed to be used together with specific modifiers within the EQ framework. As shown on the right-hand side of Figure 1, the ontology of cell phenotypes can be used to collect annotations for cell phenotypes such as fibroblast, epithelial cell and pluripotent stem cell. We can set up annotation profiles of cells, consisting of sets of EQ pairs that describe them. For example, the profile of epithelial cells includes the information that the genes/proteins Occludin, the Junctional adhesion molecule (JAM), Claudin as well as tight junctions (TJs) are 'present’, and cell membranes are 'joined’. For this purpose, we can use a number of standardized modifiers like 'present’, 'absent’, 'up’, 'moderately up’, and 'down’, which can be integrated within an ontology like PATO [13]. The quality terms used in a particular annotation profile are derived from the data being annotated, describing, e.g., a specific set of epithelial cells in a specific culture medium.

**Figure 3:**
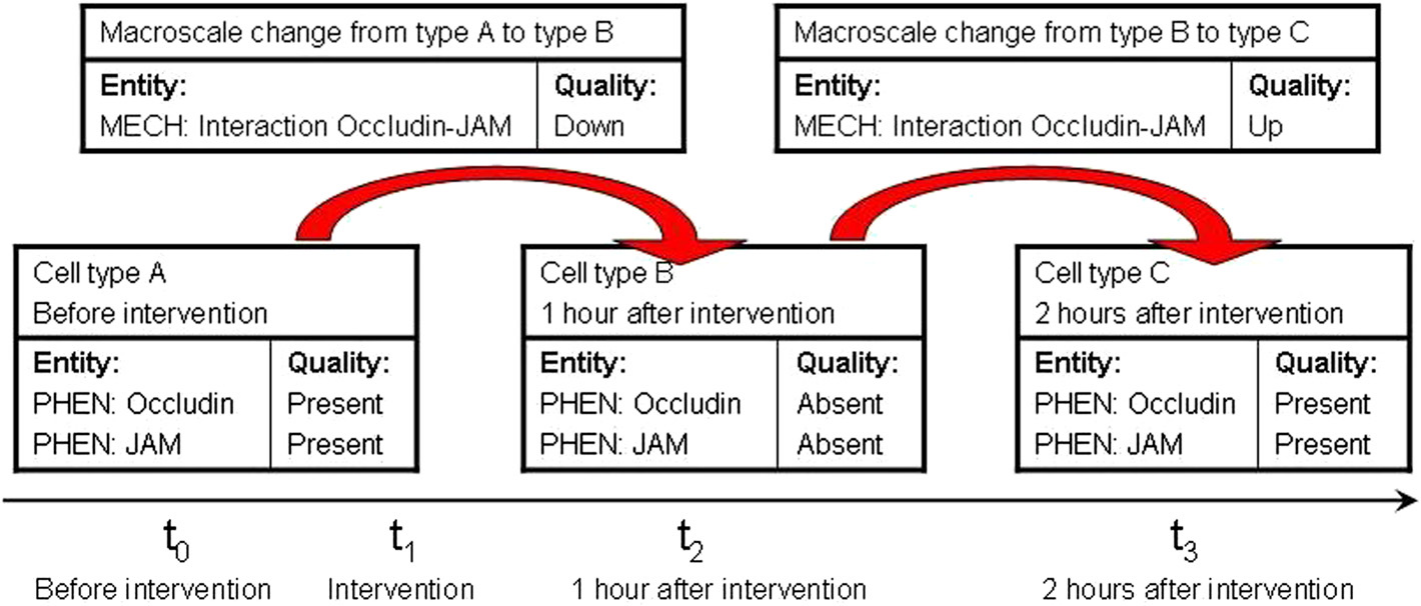
**Using EQ syntax to represent cellular dynamics.** The diagram shows how the two ontologies for cell phenotypes (PHEN) and mechanism changes (MECH) work together with the EQ syntax in order to represent the dynamics of cellular processes. It shows fictive annotation profiles for three cell types (A, B, C) and for two macroscale changes (from type A to B, and from type B to C). Each of the cell type profiles is time-stamped: A is the phenotype of the cell in question before a certain intervention, B the phenotype after 1 hour and C the phenotype after 2 hours. In each annotation profile we use, e.g., terms for molecular entities together with modifiers like 'present’ and 'absent’ to represent the participants of the microscale mechanisms that are actively ongoing in a cell at a certain point of time. The phenotypic profile of a cell will normally vary through time, and such a series of profiles can be used to adequately describe the history of a cell in an experiment. Here, we use terms for mechanisms together with modifiers like 'up’ and 'down’ in order to describe the macroscale changes of the microscale mechanism going on within the cell.

The ontology of cell mechanisms, on the other hand, is designed to be used together with modifiers like 'up’ and 'down’ in order to yield descriptions for macroscale changes of the microscale mechanisms going on within a cell ('up’ for start-up; 'down’ for shutdown). The right-hand side of Figure 2 features, e.g., the specific changes ('up’ for start-up) of the microscale mechanisms relevant for TJs, which are the macroscale changes associated with TJ formation. Within the framework of the EQ syntax, qualities help to describe these changes of the microscale mechanisms. The example hierarchy in Figure 2 reflects our example of the epithelial-mesenchymal transition (EMT) and its reversal (MET, observed during reprogramming). Within an EQ-framework, an MET can be coded using 'network of mechanisms relevant for epithelial cell’ as the entity term and 'up’ as the quality modifier; the 'network of mechanisms relevant for mesenchymal cell’ goes 'down’ simultaneously. Figure 3 shows how the ontology of phenotypes and the ontology of mechanisms can be combined in order to represent temporal dynamics.

### Annotation propagation

Both ontologies allow for annotation propagation. In [12], annotations for anatomical entities are propagated up a hierarchy of *is_a* and *part_of* relations, such that a parent receives all the annotations of its children. The rationale for this is the following. Every finger is part of a hand; hence any information about a finger is also information about some hand. Hence, whatever is explicitly annotated with “finger” is, implicitly, also about some hand. Annotation propagation makes these implicit annotations explicit via automated reasoning. In our domain, however, universal *part_of* relations are rare: As opposed to anatomical entities, molecules (like Occludin) and organelles are not usually restricted to one specific part of a cell or a specific cell, and process parts can belong to different process wholes. As a consequence of this, the mereological hierarchy cannot be used in the same way as in [12] for cell phenotypes and mechanism changes. As there is an implicit universal quantification over all instances of the first class in an assertion in an ontology description language like the Web Ontology Language (OWL) [18], we have to use *has_part* instead of *part_of.* In our example, a molecular entity like Occludin can belong to a range of cellular structures and phenotypes, while a certain cellular structure or phenotype has to possess certain molecular entities. Put in general terms, a cellular structure necessitates its essential molecular parts. That is, the whole determines the parts, and for this reason we need to use the *has_part* relation. The *has_part* relation is also appropriate for occurrents like cell mechanisms. This is because any initial temporal part of an event can happen without the event being completed. For example, not every S-phase needs to be part of a mitosis: the cell cycle can be disrupted, e.g. by the destruction of the cell that is about to divide, resulting in an S-phase that is not followed by a cell division at all. In contrast to this, every mitosis has an S-phase as one of its temporal parts. Again, we need to employ the *has_part* hierarchy, from whole processes to their necessary parts (e.g., from *Network_of_mechanisms_relevant_for_TJ* to the *Interaction_Occludin_JAM).* When employing annotation propagation, therefore, as a rule, a whole process will have a *higher* information content than its necessary parts.

### Similarity searches

The ontologies outlined above enable similarity searches across cell phenotypes and mechanism changes in analogy to [12]. In particular, we wish to estimate the similarity of cell types and of cell transitions across time. In this setting, macroscale changes are processes happening from one state at a certain time to another state at a later time. What we call microscale mechanisms are activities around a certain time, i.e. activities that we suppose to happen at some (maybe small) interval around that time. Microscale mechanisms are typically described as undirected activities (interactions), while macroscale changes are of necessity directed to a certain end state. The EQ-syntax is used to build up annotation profiles for the cell types under scrutiny. If appropriate, they will be time-stamped in order to mark how much time has elapsed after a certain intervention (e.g., “two days after intervention X”). If a macroscale change is the transition from cell type A to cell type B, it can 'inherit’ the timestamps from the annotation profiles of A and B as its start and end time, respectively (see also Figure 3).

Similarity searches may then compare, e.g., EMT/MET and reprogramming data. In simplified terms, an MET (mesenchymal-epithelial transition) [19] consists in, first, the formation of adherens junctions (AJs) and, second, the formation of tight junctions (TJs). We represent the MET as the start-up of the microscale mechanisms relevant for an epithelial cell, which has as one of its parts TJ formation that is, in turn, represented as the start-up of the mechanisms relevant for a TJ. This is the inverse of an EMT (epithelial-mesenchymal transition, which happens in development, metastasis and fibrosis). ExprEssence and related tools ([20-23]) can be employed for generating annotations about mechanism changes relevant for a certain transition by means of high-throughput data analysis, and the more mechanisms are annotated, the better we can estimate how similar biological processes are. Ultimately, any set of cell transitions can be compared (using data coded in EQ syntax) with respect to the underlying mechanisms, demonstrating the power of our approach. Our ontology design principles thus enable a kind of BLAST search in the space of annotations (for mechanisms), with similar goals such as highlighting relationships (between mechanisms, based on basic mechanisms as building blocks), and eventually estimating their evolutionary history.

**Figure 4:**
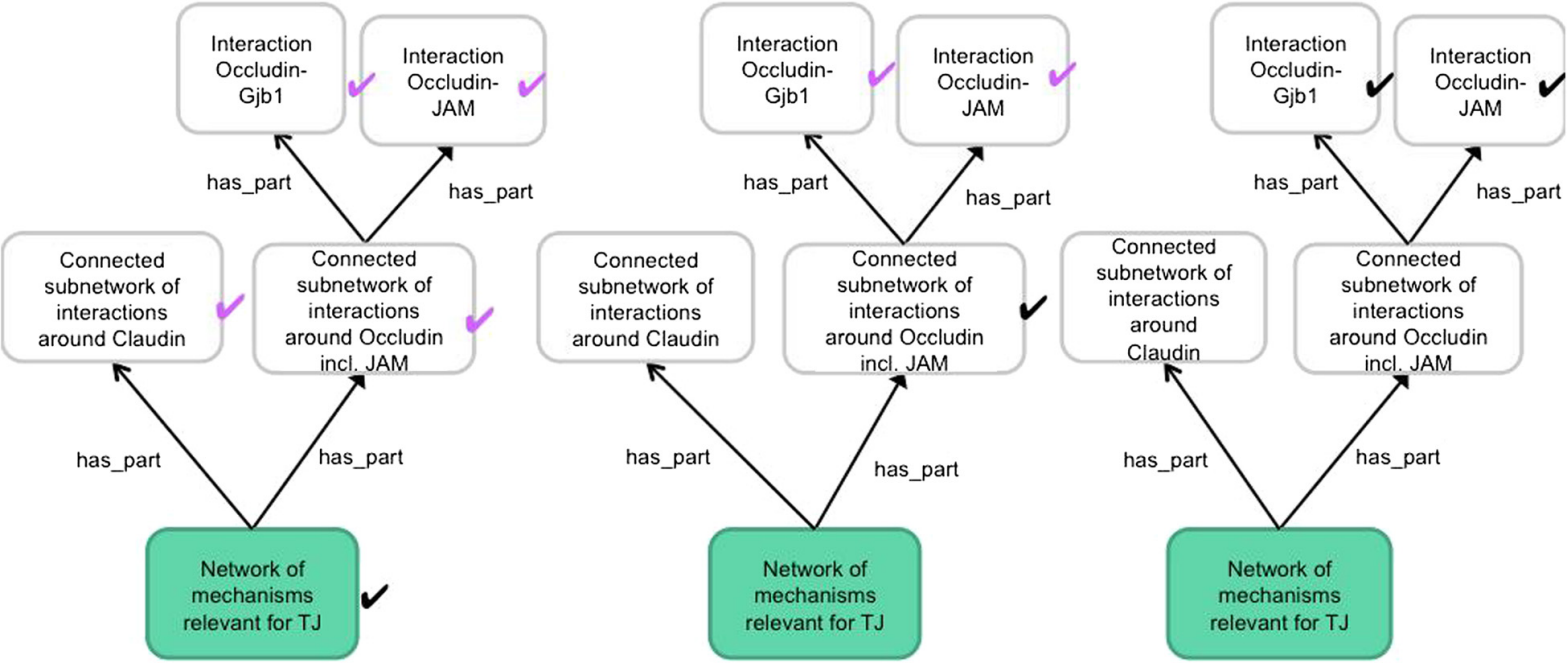
**Example of an ontology-supported similarity search.** Three possible annotation profiles derived from data on MET processes are exemplified, from left to right. Black tickmarks label mechanisms upregulated according to the data, and magenta tickmarks are inferred. After reasoning, more plausible similarity estimates become possible.

The support for similarity search that we envision is illustrated in Figure 4. From left to right, three possible annotation profiles are shown that could be derived from data on MET processes. For the sake of simplicity, the qualities considered are restricted to 'up’ and 'down’, marking upregulation of a mechanism (derived from high-throughput data or literature curation) by a black tick mark. Further assertions are then derived by automatic annotation propagation: Our ontology of cell mechanisms enables to infer (by subsumption reasoning, following the idea of [12]) the magenta tick marks. Our example suggests that similarity estimates after ontology-based reasoning are better and more reliable than without reasoning.

### Clustering of cell phenotypes

**Figure 5:**
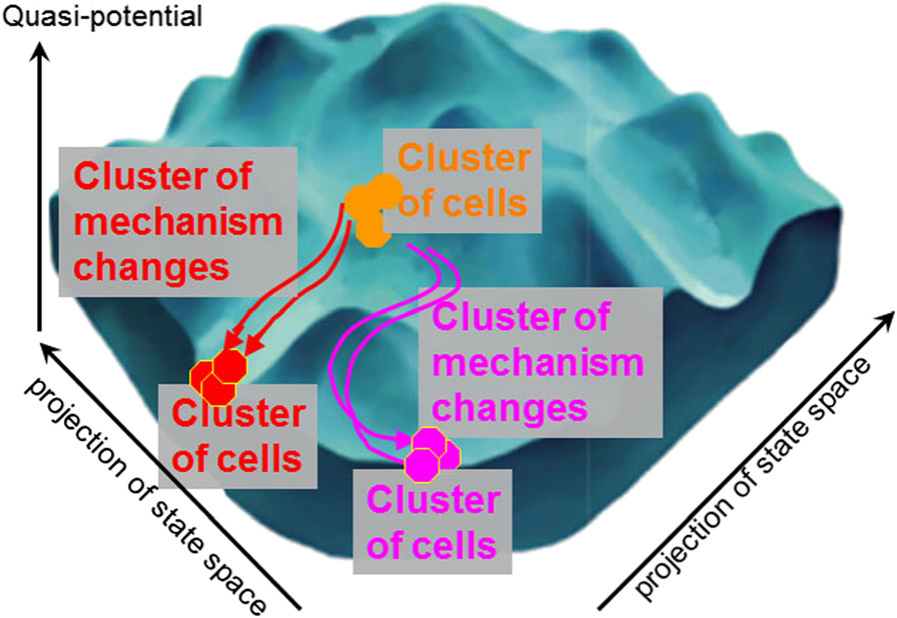
**Quasi-potential landscapes and cell phenotype and mechanism change clustering.** The “quasi-potential landscape”, adapted from [24,25], illustrates how clusters of cells (cell phenotypes) and clusters of mechanism changes (cell transitions) are related.

While our individuation criteria for cell phenotype are very fine-grained (even a tiny change in the molecular repertoire constitutes a change in phenotype), we can construct more coarse-grained cell types by clustering of cell phenotypes based on similarity, considering the presence or absence of morphological or (ultra-)structural components and of molecular entities. In addition, we can also cluster the macroscale changes that transition cells from one phenotype to another. A cluster of cells shares aspects of components and microscale mechanisms. Generally speaking, similar phenotypes correspond to similar cells, and similar mechanism changes correspond to similar cell transitions (cf. Figure 5). Thus, boundaries between clusters of cells that are 'next neighbours’ (e.g. pluripotent embryonic stem cells and epiblast stem cells) as well as between cells on opposite ends of a developmental spectrum (e.g. mesenchymal cells and epithelial cells) can be defined by clustering based on expert annotations and on bioinformatics analyses of experimental data. Clustering of mechanism changes (that is, of macroscale changes in microscale mechanisms) will in turn generate clusters of similar mechanism changes with a large distance between the clusters. The cause for this large distance then is the existence of strongly dissimilar cells.

We suggest that the improvement in similarity estimates afforded by our ontologies (see Figure 4) enables the plausible clustering of both cell phenotypes and cellular mechanisms. Then it should become possible to cluster cell phenotype and mechanism data sufficiently well to derive the clusters exemplified in Figure 5.

## Conclusion

We outlined how to design ontologies that enable to (1) formally represent cell phenotypes and mechanism changes behind cell transitions such as (re-)programming, and to (2) develop algorithms exploiting this framework, including clustering and searching for similar cell phenotypes and mechanism changes. Both ontologies support manual curation of publication data, annotation propagation and information content measurement, as well as the inclusion of results from high-throughput data analysis.

Our use of EQ-syntax allows the systematic encoding of annotation profiles of cell phenotypes and mechanism changes. The terms for both types of entities are organized in hierarchies ranging from molecular to (ultra)structural to morphological entities. Annotation profiles can then be obtained using (1) data curation from publications or by (2) high-throughput data analysis. In ontological terms, bioinformatics tools such as ExprEssence can be used as an instrument for deriving mechanistic information from high-throughput data, turning information about continuants into information about occurrents by differential analysis. The starting point for expert curation, possibly supported by text mining, must be a set of carefully selected papers.

Given a rich annotated knowledge base, existing approaches for ontology-based similarity measurements [12] can be applied to the domains of cell phenotypes and cellular mechanism changes. This would yield two important functionalities: It allows clustering of cell phenotypes (and of mechanism changes) by similarity, providing important information for an operational definition of cell phenotypes, and it allows similarity search in the spaces of mechanism changes and of cell phenotypes.

To further refine and populate the ontologies, we currently explore the option to work together with collaborators in the DFG SPP 1356 (http://www.spp1356.de) on pluripotency and cellular reprogramming, and similar initiatives, and we are looking for further collaborations. The size of the final artifacts is obviously a function of time and efforts invested in their development. While the number of relevant entities is limited for cell anatomy and cell types (several thousands), it is very large and virtually unlimited for molecular entities.

To evaluate our approach, we intent to compare similarity search results based on high-throughput data analysis only to results based on employing the ontologies integrating high-throughput data, (ultra)structural data and morphological data, and further to compare both sets of results with the expectations of domain experts. We expect that in particular the relationships between molecular events (which may be derived from filtering high-throughput data) and ultrastructural events (curated from the literature) yield improvements for similarity searches (see Figure 4). To avoid a garbage-in, garbage-out scenario, the application domain must be strictly limited, e.g. to data describing reprogramming and EMT experiments, so that the input data can all be validated by domain experts. Ultimately we envision a community-based crowd-sourcing approach.

## Competing interests

The authors declare that they have no competing interests.

## Authors’ contributions

Starting from research by GF, LJ and GF developed the ontologies, while AK and UL provided domain knowledge. LJ wrote the first version of this paper, drawing on a larger unpublished manuscript by the authors. All authors read and approved the manuscript.
